# Foot and Mouth Disease*Editorial, *The Veterinary Record*.

**Published:** 1892-03

**Authors:** 


### FOOT AND MOUTH DISEASE.*

England seems to have had a narrow escape from an invasion of Foot and Mouth Disease, but thanks to the energy displayed by the Board of Agriculture it has probably been averted. On February 4th, some cases were detected in the Islington Cattle Market, amongst animals which formed part of a cargo from Denmark. The cargo consisted of sixty-eight cattle, and much anxiety was caused by the discovery that twenty-four of them had been sold and removed to various parts of the country. The whole of these were, however, soon traced, although scattered so widely as Chatham, Colchester, Aldershot and Shorncliffe. All these animals were immediately slaughtered, as were the remainder of the cargo, and by the 7th February, the authorities were able to announce that "none of the Danish cargo remained alive." To prevent the possibility of spread by animals which had been in contact with the affected beasts, the market was closed and an order issued prohibiting the holding of all markets and of all public and private sales of cattle, sheep, goats and swine in the Metropolitan district with the exception of the foreign cattle market at Deptford, where only cattle for slaughter are admitted. Telegrams were sent to every district inspector requesting him to be alert to any danger, and the local authority also issued orders to assist in guarding every possible opening for the spread of contagion. The importation of live cattle is now forbidden from Denmark and also from the Netherlands.

It is remarkable that the Danish authorities positively assert that there is no foot and mouth disease in their country, and we know it has long been free from cattle diseases, and that their preventive laws are good and well carried out. The Danish veterinary inspectors have failed to discover any outbreak, and it seems probable that the infection was of a limited number of animals probably derived from Germany. It seems hard on the Danes to refuse their cattle when by great trouble and unremitting attention their country is free from disease, but their proximity to Germany where foot-and-mouth seems always to prevail makes it imperative that we should run no risk.

At a small estimate we may reckon that when foot and mouth disease prevailed here it caused a loss to the country of at least a million sterling per annum. In the present state of agriculture we cannot afford this, and we may congratulate the Board of Agriculture upon the efficient organization and watchful readiness which has enabled them to act so rapidly and so effectually.

---

*Editorial, *The Veterinary Record.*

